# On the Heart, and the Stimuli by Which Its Actions Are Excited; Illustrated by Experiments

**Published:** 1816-04

**Authors:** 


### *For the London Medical and Physical Journal.*

### *On the Heart, and the Stimuli by which its Actions are excited; illustrated by Experiments.*

THE heart is that viscus by whose agency in the circulation every function of the animal economy is supported; for, when its action ceases, every function is suspended. It is muscular in the human body, of a conical figure, and divided into two cavities, for the reception of the blood from the veins, and for its retention until the ventricles are enabled to receive it. Each of these cavities is provided with a vestibulum or antichamber, technically termed auricles, which are constantly supplying the ventricles with blood. Alternately with the contraction of the auricles, the ventricles having their parietes distended with this fluid, and being endowed with a considerable thickness of muscular fibre, violently contract, and by their contraction evacuate their cavities in a degree proportionate to their dimension. The contraction of the ventricles of the heart, during which time the arteries are replenished, is termed the systole; and that space of time which elapses betwixt the systole, and during which the contracted fibres are returning to a state of relaxation, is called the diastole: during the diastole the ventricles are replenished by the auricles.

But, notwithstanding, by ocular demonstration, we are enabled to ascertain that the actions of the heart thus successively take place, we are still at a loss satisfactorily to explain the mode which Nature has pursued for the production of these contractions, and the rendering the actions of this viscus regular and perpetual during life. An illustration, therefore, of this subject, is the object which I hold in view, and which, I trust, from its importance, will frame a sufficient a sufficient apology for the sacrifice I am making of your valuable pages.

The mind (or, with greater propriety I may say, the will) certainly possesses no influence either in retarding or accelerating the action of the heart. The muscular fibres entering into the composition of its structure, are consequently termed involuntary. To become acquainted, therefore, with the laws to which fibres of this denomination are obedient, is the first and indispensable measure for the completion of the task which I have undertaken.

Involuntary muscular fibres can never act but through the medium of a stimulus, and it is requisite that the operations of this stimulus upon the fibre shall be such as are capable of producing in it an uneasy state.

When, from any cause, the uneasy state of a muscular fibre is induced, it invariably contracts, and in that state remains until the renewal of the exciting principle be effected.

Consequently, the immediate object of its contraction is to relieve itself from its uneasy state; and, when this object is obtained, its relaxation follows. The bladder, having the muscular fibres of its parietes thrown into a state of uneasiness, either by their preternatural distension, or by the irritative quality of the urinary fluid, contracts, and thus removes the stimulus, by its evacuation, when the fibres relax to their natural state. The number of conditions or states, therefore, to which involuntary muscular fibres are reducable, is three:

1st, The natural or relaxed state which they at all times enjoy when in a state of rest.

2d, The uneasy state, or that which is consequent to the operations of any stimulus; and

3dly, The contracted, or that which is invariably sequential to the uneasy state.

Concerning the nature of the stimulus by the operations of which the contractions of the muscular fibres of the heart are produced, and by the cessation of whose operations they alternately relax to their natural state, as great a diversity of opinions continues to exist as with respect to any one subject in physiology.

The immediate contraction the muscular fibres of the ventricle experience upon a propulsion of a supply of blood from the correspondent auricle; their subsequent return to the easy relaxed state, when they have effected the partial evacuation of their cavity; and, lastly, the destruction to the functions of either ventricle, when, by a ligature, the flow of blood into its cavity is impeded, are weighty arguments in favour of a supposition, that the presence of the blood is actually actually necessary for inducing the contraction of these involuntary fibres. Accordingly, it has been the received opinion, that the quality of the blood is such as to throw into an uneasy state, and thus to produce the contraction of the fibres of the several cavities; but to the admission of this theory numerous are the objections, one of which only will be here mentioned, as it is sufficient to direct our decision.

Supposing that the stimulus by which the heart's action is produced to exist in the quality of the fluid, it would certainly follow (as is the case) that upon the reception of a supply of blood by either of its cavities, the fibres of which the parietes of this cavity are composed should be brought into their contracted state; and thus, by diminishing its diameter, a quantity of that fluid, proportionate to the extent of the diminution, should be evacuated. Now, as the quantity of the blood which, at every contraction of these cavities, is expelled, is only proportionate to the extent of its diametrical diminution; as the action of their parietes is never so complete as entirely to obliterate the cavity; and, as Mr. Hunter observes, that, at every systole, only two-thirds of the contents of the ventricle are expelled,—it is evident that the ventricles are never emptied; and, that, at every contraction of their parietes, they are brought into contact, for the expulsion of the part, with the remainder of the blood which was not expelled. How does it then not happen that this irritative quality of the blood does not continue its operations? that when the fibres are contracted, and forced against the residue of the stimulating fluid, the same cause does not continue to produce the same effect, so as to maintain the contracted state of the muscular fibres, instead of permitting them again to retire by relaxation to their natural easy state, and thus to transgress the most invariable law, that of continuing to contract as long as the cause of contraction continues to urge. If, therefore, this theory be admitted, it is evident that the stimulus by which the heart's action is produced does not consist in the irritative property of the circulating fluid.

Another theory which has been advanced on this subject was founded on the supposition, that, in consequence of the alternate supply of blood which the coronary arteries receive with the rest of the system, the muscular fibres of the heart were at one time in possession, and at another void of irritability, and, therefore, their states of contraction and relaxation were alternated; but this will not account for the action of the auricles being alternate with that of the ventricles, besides which, it may be ascertained by experiment

1 that that the coronary arteries receive their supply synchonous with the rest of the system.

With as little foundation has it been supposed, as the nerve (by whose influence the muscular fibres of the heart have been generally considered to be rendered capable of receiving the necessary impressions for the production of their contracted state) lies immediately betwixt the two large arteries arising from the heart, that the ventricles, at every contraction, may become so dilated as to compress the nerve, thus to paralyze for a moment the muscular structure of that viscus, and produce the relaxation of its fibres. For although, at this time, (I mean when the ventricles have contracted,) they may be permitted to be returned to their state of relaxation, having accomplished their object in the supply of the two large arteries, the action of the auricles immediately becomes necessary for the replenishment of the ventricle, and this action would be incapable of being produced, if the muscular fibres of the heart were, subsequently to contraction of the ventricle, thrown into a state of paralysis. But, supposing for a moment that the compression caused by the dilatation of the two arteries on this nerve should be capable of paralysing the ventricles alone, and the action of the auricles continue at this period unabated, how, when the quantity of the blood in the arteries is lessened, and the compression thereby renewed, is the action of the ventricle again produced? for, although, for the sake of argument, and contrary to all example, it should be allowed that the mere compression of a nerve is capable of producing so instantaneous a relaxation of the muscular fibres it supplies, how is it possible that the mere removal of that compression can be capable of inducing the immediate contraction of those fibres? for, notwithstanding the compression of a nerve will ultimately produce a paralysis of the fibres which it supplies, the removal of that compression can only restore to them the power, which, in their natural state, they should possess, of receiving the impression necessary to their contraction; and can never be productive of their contraction itself. Moreover, it is ascertained by the experiment of Borelli, that the muscular fibres of the heart are not wholly indebted to the brain as the source of its nerves, for these identical nerves, the compression of which, compatibly with this system, is considered to be productive of the temporary paralysation of its fibres, may be totally annihilated, and the action of the heart still continue unabated; but a sufficient objection to the admission of this theory is, that in the human subject, by the more remote situation of these nerves from the arteries, the former cannot be in the least influenced by compression pression in the dilatation (even if there were any, which there is much reason to doubt) of the latter.

The opinion which Mr. Hunter has entertained with respect to the nature of the stimulus which was productive of the heart's action, is explained in the following quotation:

"The alternate contraction and dilatation of the heart constitutes a part of the circulation, and the whole takes place in consequence of a necessity, the constitution demanding it; and, becoming the *stimulus*, it is rather, therefore, the want of repletion, making a *negative impression* on the constitution, than the immediate application of something to the heart itself.

"This we see wherever a constant supply or some kind of aid is wanting, in consequence of some action: thus we have as regularly the stimulus for respiration,—no sooner is one act performed than an immediate demand takes place for another; and thus we have the stimulus of want of food, which is regularly produced when in health."

Now, with all due deference to the superior abilities of Mr. Hunter, I am of opinion that his mode of reasoning is rather calculated to conceal than to explain the real cause by which the heart's action is produced.

We know that every other function which is performed by the action of involuntary muscles, can only be effected by those muscles being reduced to an uneasy state, and we are unacquainted with the possibility of this state being produced in any fibres of the involuntary determination, but by the action of some stimulus capable of being topically applied to their surface, or of so operating, for instance, as, by extension, to be productive of an uneasy sensation. And, although Mr. Hunter observes, that the stimulus for respiration is regularly produced, and no sooner is one act performed than an immediate demand for another takes place, yet it may be perceived that it is not the mere demand from the constitution which is capable of becoming the stimulus to this effect, but that it is the state of uneasiness or oppression, which, if felt in the chest, in consequence of the lungs becoming loaded with blood unfit for circulation, by which respiration is invited, and by compliance with which effort the oppression is removed. Neither is it reasonable to suppose, upon a due consideration of the effects which are produced, that hunger is simply and nothing more than a negative impression made in consequence of a demand from the constitution: did hunger never exceed the limits of the necessity or demand from the constitution for repletion, few would be the number of our apoplectic patients, and still fewer would be those who, during a state of plethora and and fullness of blood, have their regular calls for food, whilst no demand on the part of the constitution can exist. It is, therefore, reasonable to suppose that the immediate cause of hunger is the effect of some topical impression made upon the alimentary canal, either by the alteration of its parietes during the continuance of the peristaltic motion, in consequence of its emptiness, or the action of the gastric or enteric juice upon the living matter.

Notwithstanding the refutation which the very arguments that, for the establishment of this doctrine, Mr. Hunter has produced, let us, *argumenti causâ*, suppose, the demand which the constitution is perpetually making for the repletion of the vascular system, to be the stimulus by which the action of the heart is regularly produced.

If the demand which the constitution makes upon the heart be the stimulus which is productive of its action, it follows, that in proportion to the demand or necessity of the constitution, so will be the stimulus; and does it not also follow, that, in proportion to the demand or stimulus, so should be the action? Now, as the quantity of blood which, at every systole of the ventricles, cannot be subjected to much variety from the usual determinate extent of its diametrical diminution, it consequently happens that wherever an extraordinary demand for a repletion of the circulating system takes place on the part of the constitution, the only method by which this viscus can become equal to the demand made upon it, is by accelerating its action. It cannot but be evident to every considerate person, who for a moment reflects upon the necessity of a supply of blood for a due performance of every function, that the demand for this supply would be proportionably greater with the parts to be supplied; and, consequently, in the larger animals, than those of inferior magnitude; and, *consideratis considerandis*, that the action of the heart will become more frequent, being unable to meet the necessity but by the increase of its rapidity.

Supposing, then, the two foregoing points to be established, viz. that, in proportion to the magnitude of the parts to be supplied, so will be the demand from the constitution for repletion augmented; and 2dly, that, as the extent of the dimensions of the ventricles is subjected but to little variety, so as materially to augment or diminish the evacuations which are made from their cavities at every systole, that every extraordinary demand being made upon the heart from the constitution can only be obeyed by the increased velocity of its contractions,—we will proceed to the analysis of the validity of Mr. Hunter's opinion.

The heart of a horse does not contract more frequently than 36 times in a minute; whilst that of a man, *cæteris paribus*, seldom fails to exceed 70 or 72.

In a man, eight feet high, the pulse does not exceed 60; whilst in one who is only four feet in height, the heart acts 75 times in the space of a minute.

Now, as the magnitude of the parts to be supplied in the horse must certainly treble those of an ordinary sized man, it follows that the demand from the constitution is three times as great; the stimulus, consequently, being trebly extensive, the action of the heart should be three times as frequent. The number of times, therefore, which the heart would be necessitated to act in the space of a minute, to overcome the necessity or demand from the constitution, in the case of the horse, instead of being only 36, should equal three times that of a man, being 72, which would amount to 216.

In inflammatory fevers, does the action of the heart never take place but from the constitutional demands operating as the stimulus? In plethora, when the demand from the constitution on the heart must be very sparing, does it often happen that the pulse becomes less frequent? From the due consideration of the preceding objections, and the incapacity of the supporters of such a doctrine to account for the alternate action of the auricles and ventricles, the increase of rapidity of the heart's action, when, by an augmented supply of blood, its irritability is increased; and the cessation which its action experiences, when, from a deficiency, its irritability is diminished, as in ossification of the coronary arteries, which seem to imply that topical impression must necessarily be made upon the parietes of the ventricles themselves to produce their contraction, the fallacy of Mr. Hunter's doctrine seems implied.

**[To be continued in the next Number, in which will be introduced the outlines of another System, &c. &c. with an experimental illustration of the subject.]**

---

*For the London Medical and Physical Journal.*

*Hints to the Editors;* by A. Z.

